# A spatio-temporal model reveals self-limiting Fc*ɛ*RI cross-linking by multivalent antigens

**DOI:** 10.1098/rsos.180190

**Published:** 2018-09-26

**Authors:** Md Shahinuzzaman, Jawahar Khetan, Dipak Barua

**Affiliations:** Chemical and Biochemical Engineering, Missouri University of Science and Technology, Rolla, MO 65409, USA

**Keywords:** Brownian dynamics, spatio-temporal modelling, immunoreceptor, cell signalling

## Abstract

Aggregation of cell surface receptor proteins by multivalent antigens is an essential early step for immune cell signalling. A number of experimental and modelling studies in the past have investigated multivalent ligand-mediated aggregation of IgE receptors (Fc*ɛ*RI) in the plasma membrane of mast cells. However, understanding of the mechanisms of Fc*ɛ*RI aggregation remains incomplete. Experimental reports indicate that Fc*ɛ*RI forms relatively small and finite-sized clusters when stimulated by a multivalent ligand. By contrast, modelling studies have shown that receptor cross-linking by a trivalent ligand may lead to the formation of large receptor superaggregates that may potentially give rise to hyperactive cellular responses. In this work, we have developed a Brownian dynamics-based spatio-temporal model to analyse Fc*ɛ*RI aggregation by a trivalent antigen. Unlike the existing models, which implemented non-spatial simulation approaches, our model explicitly accounts for the coarse-grained site-specific features of the multivalent species (molecules and complexes). The model incorporates membrane diffusion, steric collisions and sub-nanometre-scale site-specific interaction of the time-evolving species of arbitrary structures. Using the model, we investigated temporal evolution of the species and their diffusivities. Consistent with a recent experimental report, our model predicted sharp decay in species mobility in the plasma membrane in response receptor cross-linking by a multivalent antigen. We show that, due to such decay in the species mobility, post-stimulation receptor aggregation may become self-limiting. Our analysis reveals a potential regulatory mechanism suppressing hyperactivation of immune cells in response to multivalent antigens.

## 1. Introduction

Cross-linking of cell surface receptor proteins by antigens is an essential early step for the activation of all immunoreceptor signalling pathways. In the past, both experimental and modelling studies investigated multivalent ligand-mediated cross-linking of IgE receptors (Fc*ɛ*RI) in the plasma membrane of basophil or mast cells [1–8]. Synthetic multivalent ligands have been used to understand the correlation between membrane clustering of Fc*ɛ*RI and cellular histamine release or degranulation [8–13]. However, the mechanistic understanding of multivalent ligand-Fc*ɛ*RI assembly in the cell membrane remains incomplete.

A number of experimental studies have found that stimulation of cells with a synthetic multivalent ligand forms finite-sized Fc*ɛ*RI clusters in the plasma membrane [8,14–16]. This observation contradicts model-based analysis [3,7]. On the contrary, an early theoretical model by Goldstein & Perelson [3] predicted that stimulation of a bivalent Fc*ɛ*RI with a trivalent ligand may lead to a condition where all the receptors can be incorporated into a single large complex (superaggregate or gel). They postulated that such superaggregate formation might enable a cell to be hyper-responsive against infection. Recently, using a stochastic model, Monine *et al.* [7] also showed similar phenomenon in Fc*ɛ*RI aggregation. Lately, Mahajan *et al.* [8] reported a model similar to the model of Monine *et al.* [7]. However, this model imposed a condition on receptor cross-linking to prohibit superaggregate formation.

A common caveat of these earlier models was that the models were non-spatial and hence lacked the ability to incorporate the spatio-temporal effects, such as membrane diffusion, steric collision and geometry of the dynamically evolving species (ligand and receptor molecules and their complexes). We were interested in investigating whether a spatial model could explain the reported behaviour of Fc*ɛ*RI aggregation. Recent experimental studies by Shelby *et al.* [16] and Andrews *et al.* [14,15] indicate that the diffusion of multivalent antigen-cross-linked receptors falls sharply upon stimulation of mast cells. In particular, the analysis of Andrews *et al.* [14] suggests that the multivalent ligand cross-linked receptor cluster display reduced mobility through the actin network of the cell plasma membrane. The question we wanted to answer is whether such decrease in the receptor mobility could ultimately dictate receptor cross-linking and the size distribution of Fc*ɛ*RI aggregates in the plasma membrane.

To develop a spatial model, we needed a computationally efficient simulation framework that can efficiently capture the site-specific interactions of the molecules and complexes at reasonably high resolutions. A typical molecular dynamics (MD) simulation was deemed infeasible because of the timescale and spatial domains of our interests. Therefore, we adopted a Brownian dynamics (BD)-based approach. To leverage computation, we implemented a time-adaptive feature in the BD simulation algorithm based on our recent work [17]. We integrated the time-adaptive BD algorithm with an agent-based framework, where we used spatial graphs to define the coarse-grained structures (site-specific features) of the trivalent ligand and bivalent Fc*ɛ*RI molecules in a two-dimensional plane (cell membrane).

Using the model, we investigated the effects of membrane diffusion on the interaction of dynamically evolving ligand-receptor complexes. Consistent with [16], our model indicated that there could be a rapid decay of species diffusion in the membrane upon stimulation by a multivalent antigen. We showed that the dynamics of this decay is correlated to the level of receptor density in the cell membrane. We compared the predictions of our spatial model with a non-spatial model, which we developed based on the models in [7,8]. The spatial model predicted finite-sized receptor clusters distributed over a broad range of sizes. By contrast, the non-spatial model revealed a single superaggregate incorporating approximately all receptors. We found that when the diffusion constant in the spatial model was made time-invariant and species-independent, it also predicted superaggregate formation like the non-spatial model. These results underscore a limitation of the non-spatial models, where a bimolecular association rate constant accounts for the ligand-receptor assembly while ignoring the time-evolving size and geometry of the species. Our analysis indicates that cell surface receptor aggregation could be self-limiting due to the growing aggregate sizes and concurrent reduction in their mobilities. We conclude that such a self-regulatory mechanism may serve to contain hyperactive cellular responses.

## 2. Material and methods

Our spatio-temporal model was based on a trivalent ligand and a bivalent receptor molecule. The model resembles the trivalent ligand and bivalent Fc*ɛ*RI systems studied in [8]. The model is developed using the agent-based approach combined with a time-adaptive BD simulation algorithm. Below we provide the details of the model and the BD algorithm.

### 2.1. Spatio-temporal model of trivalent ligand-bivalent receptor interaction in the cell membrane

**Figure 1.**
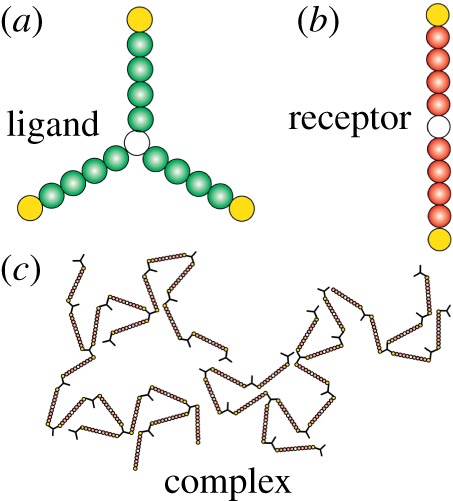
Illustration of spatial graphs. (*a*) A trivalent molecule. The white unfilled circle at the centre defines the molecule centre, and each yellow circle represents a reaction centre. (*b*) A bivalent receptor molecule. The unfilled circle at the centre defines the molecule centre, and each yellow circle represents a reaction centre. (*c*) A ligand-receptor complex.

In our model, ligand and receptor molecules are described by agents or software objects. We consider a two-dimensional rectangular plane to define the plasma membrane, where the molecules can diffuse and mediate site-specific interactions. Using an approach similar to [18], we use spatial graphs to describe the ligand and receptor molecules (figure 1). A spatial graph is a collection of small circles, which we refer to as subunits. The spatial orientation of the subunits defines the structure of a molecule and its site-specific features, such as binding domains or motifs.

We define a ligand molecule based on the synthetic trivalent ligand described in Mahajan *et al.* [8]. The molecule has three identical binding arms each of which is approximately 17 Å long. The binding arms are symmetrically spaced, i.e. the angle between two adjacent arms is 120°. We consider a single subunit of radius 1.5 Å to define the centre of the molecule (figure 1*a*). We define each binding arm by five adjacent subunits each of which is also 1.5 Å in radius. Thus, the distance between the molecule centre to the tip of each binding arm is approximately 16.5 Å. We consider each binding arm to have a reaction centre that can form a bond with a complementary region of a receptor molecule. In each binding arm, we designate the subunit at the tip as the reaction centre (yellow circles in figure 1*a*).

We model the bivalent Fc*ɛ*RI by a graph containing two identical binding arms 180° apart (figure 1*b*). Reports indicate that the approximate Fc*ɛ*RI radius is 46–51 Å [19]. We consider each binding arm to have five identical subunits each of which is also 5 Å in radius. Thus, the length of each binding arm is 50 Å. We consider a molecule centre separating these two arms. A single subunit of 5 Å radius defines the molecule centre. Similar to a ligand molecule graph, each binding arm contains a reaction centre at the tip (yellow circles in figure 1*b*).

In the model, when the reaction centres of a ligand and a receptor molecule graph come within a close proximity (a predefined short distance that we call reaction layer), they may form an (implicit) bond. The bond holds the two molecules together in a complex. Similarly, a complex may form larger complexes through association with other molecules or complexes via their unoccupied reaction centres. Figure 1*c* illustrates a complex containing multiple ligand and receptor molecules.

### 2.2. Time-adaptive Brownian dynamics simulation algorithm

We use a time-adaptive BD algorithm to simulate species diffusion in the two-dimensional membrane. The time adaptive feature is included to accelerate computation, especially in the dilute regimes. The algorithm is based on our recent work [17]. It selects the time step sizes adaptively to facilitate computation while capturing the site-specific interactions of the species at the sub-nanometre resolution.

#### 2.2.1. Lateral and rotational diffusion of species

In the BD algorithm, we consider both lateral and rotational diffusion for the spatial graphs representing the species (molecules and complexes). Because the size and structure of these graphs evolve dynamically due to their site-specific assembly and dissociation, their lateral and rotational diffusivities also change with time. In every BD step, a species graph is translated or rotated as a rigid body over time step Δ*t*. We assume that the translation and rotation of a graph occur with respect to its centre of mass. Both types of diffusivities of a graph depend on its instantaneous size. We determine the centre of mass (*C*) and the size (*R*) of a graph as explained next.

**Figure 2.**
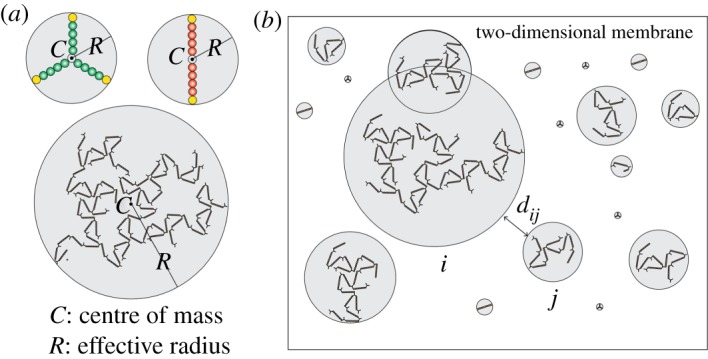
Disc representation of molecules and complexes. Each molecule and complex graph is contained by a hypothetical disc (grey circles). The centre of each disc is located at the centre of mass of the corresponding graph. The radius of the disc is the shortest distance that allows the disc to fully encompass the structure of the graph. The radius of the disc represents the effective radius of the corresponding species. (*a*) The centre of mass (*C*) and effective radius (*R*) of a ligand molecule, a receptor molecule and a complex. (*b*) Molecule and complex graphs in a two-dimensional plane (cell membrane). The variable *d*_*i,j*_ represents the distance between a pair of discs *i* and *j*. A negative *d*_*i,j*_ implies the overlap between the two discs. Such overlap is permitted as long as the structure defined by the graphs do not collide or conflict.

We consider a weight associated with each subunit of a molecule. The receptor molecule has a total of 11 subunits, whereas the ligand molecule graph has a total of 16 subunits. We assign each receptor subunit a mass of 1 (arbitrary unit). Comparing the volume of a ligand subunit with that of a receptor subunit, we assign each ligand subunit a mass of 0.027. Because these molecule graphs have symmetric structures, their centre of mass corresponds to the molecule centre (figure 2*a*). However, for a multimolecular complex, the centre of mass depends on the instantaneous spatial organization of the subunits (figure 2*a*).

We define the size of a graph by its effective radius *R*. To calculate *R*, we envision a hypothetical disc encompassing each species graph (figure 2*a*). The centre of the disc is located at the centre of mass of the graph. We consider *R* to be the distance from the centre of mass to the farthest point of the graph. For example, if (*x, y*) represents the centre of mass of a graph, and ($x_{i},y_{i}$) represents the centre of any subunit *i*, then $R=\max\left\{\sqrt{{\left(x_{i}-x\right)}^{2}+{\left(y_{i}-y\right)}^{2}}+r_{i}\right\}$, where *r*_*i*_ is the radius of subunit *i*.

We relate the diffusion constant of a species to its size by the following expression: $D\propto 1/R^{\alpha }$. Here, the exponent *α* is a tunable parameter in the model. Setting *α* = 0 makes *D* a constant (independent of time or species). This condition corresponds to a non-spatial model, where the single-site ligand-receptor association is governed by a constant rate parameter typical for any bimolecular reaction. At *α* = 1, *D* evolves naturally (based on the Einstein–Stokes equation) with the size of a graph. Finally, at *α* > 1, the model captures an arbitrarily large decrease of *D* of a growing species.

For an isolated receptor molecule, whose size (and hence diffusion) is time-invariant, we consider a baseline diffusion constant, $D_{0}=10^{4}\;{\mathrm{nm}}^{2}\;s^{-1}$ [20]. For any other species, which includes an isolated ligand molecule, we consider $D=D_{0}{\left(R_{0}/R\right)}^{\alpha }$, where *R* and *R*_0_ represent the effective radius of the species and an isolated receptor molecule, respectively.

During each BD time step Δ*t*, we advance each graph as a rigid body by a distance $l=\sqrt{4D\Delta t}e$, where *D* is the lateral diffusion constant of the graph and ***e*** is a unit vector in a random direction. After the lateral translation, we rotate the graph around the centre of mass *C* by an angle $\phi =(\sqrt{3}/2\sqrt{2})(l/R)$. The direction of rotation is chosen randomly clockwise or anti-clockwise in each time step. The expression for *ϕ* is derived considering $l=\sqrt{4D\Delta t}$ and $\phi =\sqrt{2D_{r}\Delta t}$, where *l* and *ϕ*, respectively, represent the lateral and angular displacements over time Δ*t*, *D* represents the lateral diffusion constant, and *D*_r_ represents the rotational diffusion constant. Again, *D* and *D*_r_ can be related based on Einstein–Stokes equation, $D=K_{B}T/6\pi \mu R$ and $D_{r}=K_{B}T/8\pi \mu R$, where *K*_B_ is the Boltzmann constant, *T* is temperature and *μ* is the membrane viscosity.

#### 2.2.2. Time-adaptive advancement of molecule and complex graphs

In each BD step, we calculate the time step Δ*t* adaptively. The adaptive Δ*t* is computed based on the inter-species distances *d*_*i,j*_s (figure 2*b*). If *d*_*i,j*_ for any pair of graphs (*i, j*) is small (i.e. an encounter between a pair of graphs is likely), Δ*t* is chosen small. On the other hand, if *d*_*i,j*_ is large for all unique pairs (*i, j*), Δ*t* is chosen large to accelerate computation.

To calculate the appropriate size for Δ*t*, we first compute *d*_*i,j*_ for all unique pairs of (*i, j*) for *i* ≠ *j*. As illustrated (figure 2*b*), $d_{i,j}>0$ implies that the corresponding discs are at non-overlapping positions. On the other hand, $d_{i,j}<0$ implies that the discs are at overlapping positions. We compute the smallest inter-species distance, $d_{\min}=\min\{d_{i,j}\}$ for i≠j. If $d_{\min}>0$, then none of the discs in the system should be at an overlapping position. On the other hand, if $d_{\min}\leq 0$, at least one pair of discs should be at overlapping positions.

If $d_{\min}\leq 0$, i.e. at least one disc pair is overlapping, a collision or reaction could be imminent. To capture such events more accurately, we consider a fine resolution Δ*t* under such conditions. We set the time step, $\Delta t=l_{\min}^{2}/4D_{\max}$, where $l_{\min}$ is the lower bound on species jumps in our simulation, and $D_{\max}$ is the diffusion coefficient of the fastest (smallest) disc in the system. We consider $l_{\min}=1\;\mathrm{nm}$. Thus, all discs advance by less than or equal to 1 nm when the probability of a collision or reaction is high.

If $d_{\min}>0$, i.e. none of the discs are at overlapping positions, we calculate the shortest possible time (Δ*t*_s_) that can potentially lead to an overlap between any two discs: $\Delta t_{s}=\min\{d_{i,j}^{2}/4{\left({\sqrt{D}}_{i}+{\sqrt{D}}_{j}\right)}^{2}\}$. We then check if Δ*t*_s_ is too small to consider for computational efficiency. We compare it with a defined lower bound on time step size, $\Delta t_{\min}=10^{-5}\;s$. If $\Delta t_{s}\leq \Delta t_{\min}$, we disregard Δ*t*_*s*_ and set time step $\Delta t=l_{\min}^{2}/4D_{\max}$. However, if $\Delta t_{s}>\Delta t_{\min}$, we check if any specie may advance by a distance greater than $l_{\max}=10\;\mathrm{nm}$, where $l_{\max}$ is an upper bound imposed on the jump size of species. To ensure that no species violates this upper bound, we evaluate if $\sqrt{4D_{\max}\Delta t_{s}}\geq l_{\max}$. If this condition is satisfied, we set time step $\Delta t=l_{\max}^{2}/4{\left({\sqrt{D}}_{\max}+{\sqrt{D}}_{\max}\right)}^{2}=l_{\max}^{2}/16D_{\max}$ so that the jump remains within the upper bound. Otherwise, we set time step $\Delta t=\Delta t_{s}$.

#### 2.2.3. Collision, binding and dissociation

A collision may occur if two graphs occupy conflicting positions as a result of a move. In such a possibility, we simply reject the move of the particular graph while advancing others. In the model, a binding may occur if a ligand reaction centre and a receptor reaction centre fall within a predefined small distance (reaction layer) *l*_r_ [21]. For each such pair of reaction centres, we consider a probability of bond formation, $k_{f}\leq 1$. We draw a random number between 0 and 1. If the random number is smaller than *k*_f_, we consider the formation of an (implicit) bond between the reaction centres. In our simulations, we assign *l*_r_ = 5 Å, a distance comparable to the radius of a receptor reaction centre. In the model, either *l*_r_ or *k*_f_ value can be tuned to make the system reaction-limited or diffusion-limited.

After the bond formation between the reaction centres, we treat the two associated graphs as a single (larger) graph. As in the existing non-spatial models [3,7,8], we prohibit intra-complex binding (ring formation). A bond can form only if the reaction centres belong to two distinct species (separate molecules or complexes).

We consider a dissociation rate constant *k*_*r*_ for each ligand-receptor bond. Thus, the lifetime of each bond is exponentially distributed with mean lifetime $\lambda =1/k_{r}$ s. At the end of each BD step, we calculate the probability of dissociation of each ligand-receptor bond. We draw a random number between 0 and 1. If the random number is smaller than $1-\exp(-k_{r}\Delta t)$, where Δ*t* is the latest time step, we assume the bond is broken and the graph is separated into two smaller graphs. In the next step, the separated graphs may move away from each other because of their diffusion. Alternatively, they may recombine based on the probability *k*_f_ to form a complex again. We consider $k_{r}=0.001\;s^{-1}$ [8]. Setting this parameter to zero makes ligand-receptor binding irreversible. Table 1 summarizes the default values used in the model.

**Table 1.** Default parameter values used in the spatial model.

parameter	comment
*A* = 1000 × 1000 nm	dimension of the plasma membrane domain
*n*_R_ = 80 molecules μm^−2^	density of receptor molecules
*n*_L_ = 80 molecules μm^−2^	density of ligand molecules
*N*_Rsite_ = 2	number of binding arms in a receptor molecule (bivalent)
*N*_Lsite_ = 3	number of binding arms in a ligand molecule (trivalent)
*R*_*R*_ = 55 Å	effective radius of a receptor molecule
*R*_L_ = 16.5 Å	effective radius of a ligand molecule
*D*_R_ = 10^4^ nm^2^ s^−1^	diffusion constant for an isolated receptor molecule
*D*_L_ = 10^4^[*R*_R_/*R*_L_]^α^ nm^2^ s^−1^	diffusion constant for an isolated ligand molecule
*D*(*t*) = 10^4^[*R*_R_/*R*(*t*)]^α^ nm^2^ s^−1^	diffusion constant for a complex
*α* = 1	exponent relating diffusivity and size: *D* ∝ 1/R^*α*^
*l*_r_ = 5 Å	reaction layer around each reaction centre
*k*_f_ = 1	bond formation probability when two reaction centres are within *l*_r_
*k*_r_ = 0.001 s^−1^	bond dissociation constant

### 2.3. Non-spatial trivalent ligand-bivalent receptor model

We developed a corresponding non-spatial model using the network-free kinetic Monte Carlo (KMC) approach [22]. The model is based on the earlier models in [7,8]. The purpose of this non-spatial model was to compare it with the spatial model predictions. Although written in C++, for convenience, we explain this model using the standard notations of BNGL [23], a rule-based model specification language.

The model can be described by the BNGL rule in equation (2.1). The rule defines the single-site binding and dissociation between a trivalent ligand molecule L(a,a,a) and a bivalent receptor molecule R(b,b). The ligand molecule contains three identical reaction centres (binding sites) denoted as *a* and the receptor molecule contains two identical reaction centres denoted as *b*. We use non-spatial graphs to define these molecules.

2.1 $$L(a)+R(b)\leftarrow \to L(a!1).R(b!1)\;k_{\mathrm{on}},\;k_{\mathrm{off}}$$

The rule above specifies the binding and dissociation between a pair of ligand and receptor reaction centres. In the rule, the remaining reaction centres of the two molecules are not specified. These unspecified reaction centres are wildcards, meaning those could either be free or connected to other molecules. In the rule, the symbol ! followed by a matching number (1 in this example) represents a bond connecting the reaction centres. The parameter *k*_on_ represents the rate constant for the single-site bimolecular binding (forward reaction). The parameter *k*_off_ represents the rate constant for the unimolecular bond dissociation (reverse reaction). The values of these rate parameters are independent of the remaining reaction centres that are not specified in the rule. These parameters are also independent of time and size of the species involved. Thus, at any time during the course of a simulation, the above rule gives rise to an arbitrary number of elementary reactions depending on the number of free reaction centres in different molecules and complexes.

It should be noted that the earlier models in [7,8] consider an additional rule to describe ligand recruitment from the solution (extracellular space) to the plasma membrane. In our model, we eliminate this additional step to make the non-spatial model consistent with the spatial model. This simplification is based on our assumption that the extracellular solution is well-mixed and diffusion there is fast compared to diffusion in the cell membrane.

We set $k_{\mathrm{off}}=0.001\;s^{-1}$ [8] (the corresponding parameter in the spatial model *k*_*r*_ has the same value). As discussed in the Results section, by comparing the non-spatial model and spatial model predictions, we determined $k_{\mathrm{on}}=1.667\times 10^{4}\;{\mathrm{nm}}^{2}\;s^{-1}$. Table 2 summerizes the default parameter values used in the model.

**Table 2.** Default parameter values used in the non-spatial model.

parameter	comment
$n_{R}=80$ molecules	number of receptor molecules
$n_{L}=80$ molecules	number of ligand molecules
$N_{\mathrm{Rsite}}=2$	number of binding sites in a receptor molecule
$N_{\mathrm{Lsite}}=3$	number of binding sites in a ligand molecule
$k_{\mathrm{on}}=1.66\times 10^{4}\;{\mathrm{nm}}^{2}\;s^{-1}$	forward rate constant for single-site binding (this work)
$k_{r}=0.001\;s^{-1}$	reverse rate constant for single-site dissociation

#### 2.3.1. Code implementation

Both the spatial and non-spatial models were written in Object-Oriented C++. The source codes for both models are included in the electronic supplementary material (archived folders named ‘spatial_model.tar.gz’ and ‘nonspatial_model.tar.gz’, respectively). Detailed instructions for the installation and execution of simulations are provided in a file named ‘README.txt’ in each folder.

## 3. Results

### 3.1. Effect of receptor density on ligand-receptor assembly

**Figure 3.**
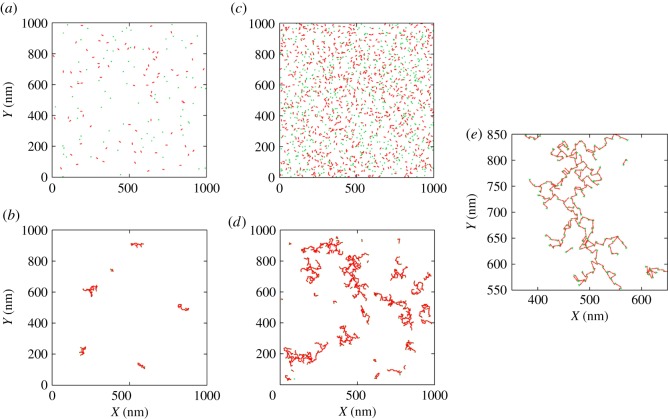
Simulation snapshots of ligand and receptor clusters in the cell membrane. (*a*) $n_{L}=n_{R}=80\;\mathrm{molecules}\;\mu m^{-2}$, t=0 s. (*b*) $n_{L}=n_{R}=80\;\mathrm{molecules}\;\mu m^{-2}$, t=30 s. (*c*) $n_{L}=n_{R}=800\;\mathrm{molecules}\;\mu m^{-2}$, t=0 s. (*d*) $n_{L}=n_{R}=800\;\mathrm{molecules}\;\mu m^{-2}$, t=30 s. (*e*) A zoomed-in region of (*d*).

We first used the spatial model to investigate ligand-receptor assembly at different levels of receptor densities in the cell membrane. Reportedly, Fc*ɛ*RI copy number in mast cells may vary between 10^4^ and 10^6^ molecules per cell [24]. We wanted to see how such variations in the receptor expression may affect receptor cross-linking. Figure 3 shows several snapshots from our simulations. As indicated in the figure, in one case we considered a relatively modest level of receptor density, $n_{R}=80\;\mathrm{molecules}\;\mu m^{-2}$ (figure 3*a*–*b*). In the other case, we considered a 10-fold higher receptor density, $n_{R}=800\;\mathrm{molecules}\;\mu m^{-2}$ (figure 3*c*–*e*). These densities correspond to 10^5^ and 10^6^ receptor molecules per cell, respectively, assuming a spherical cell of 10 μm radius. In both cases, we kept receptor to ligand ratio 1. In the cell membrane, the ligand density cannot exceed twice the Fc*ɛ*RI density because a single Fc*ɛ*RI can bind at most two ligand molecules at a time. By choosing this ratio, we allowed adequate receptor cross-linking.

Figure 3*a*,*c*, respectively, shows snapshots corresponding to the above two receptor densities at t=0. Figure 3*b*,*d* shows corresponding snapshots at t=30 s after the ligand addition. Both cases indicate finite-sized receptor aggregation at t=30 s. Not surprisingly, the higher density case shows relatively larger aggregate formation. However, the formation of larger aggregates also came at the expense of lesser mobilities. If continued longer, the aggregates in both cases would further grow but at relatively slow rates. The dynamics in the high-density case would be rather slower due to the more sluggish motion of the larger aggregates. In these simulations, we set *α* = 1. Therefore, the diffusivities of the aggregates changed naturally (based on the Einstein–Stokes equation). As discussed in the next section, even such natural decrease in diffusion led to a sharp decay in their mobility for further growth and aggregation.

### 3.2. Temporal evolution of species diffusivity

**Figure 4.**
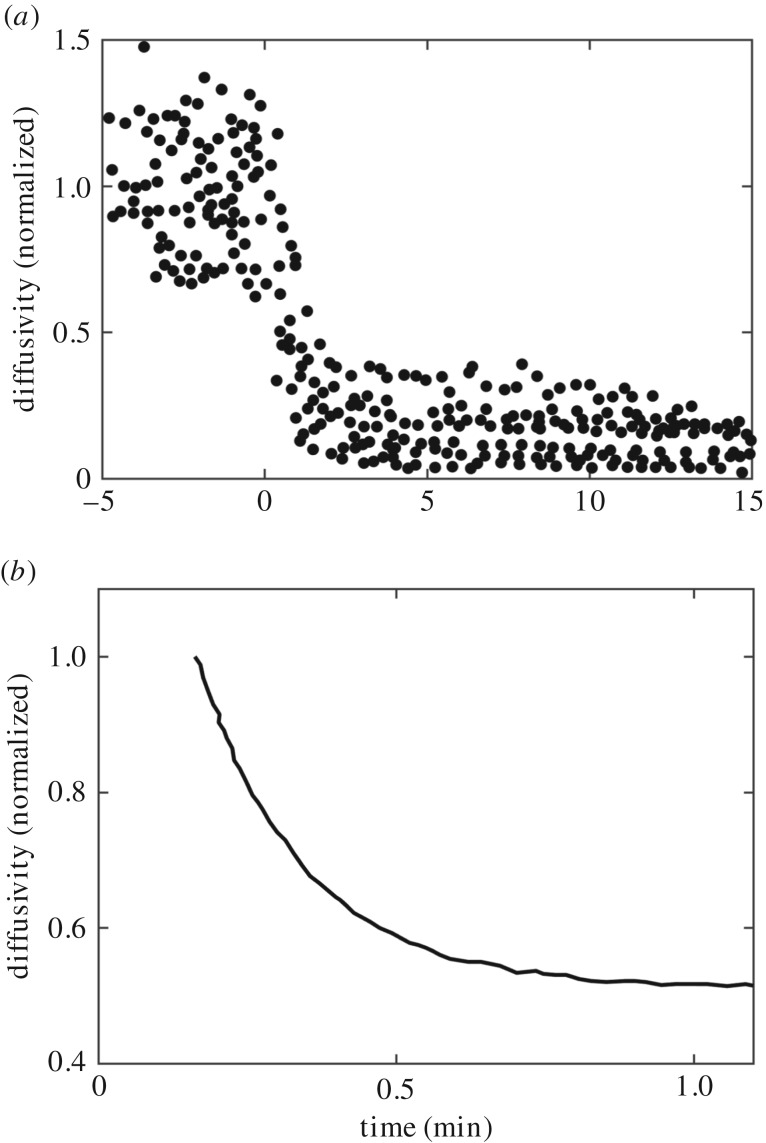
Experimental data adapted from published literature. In (*a*,*b*), the *Y*-axis represents normalized diffusion coefficient of species in the plasma membrane of rat basophil leukaemia (RBL)-2H3 cells. The *X*-axis represents stimulation time. Time zero is the time of addition of a multivalent ligand. (*a*) Fig. 2C of Shelby *et al.* [16]. (*b*) Fig. 6c of Andrews *et al.* [14].

Recently, Shelby *et al.* [16] and earlier Andrews *et al.* [14,15] investigated post-stimulation change in the diffusion of Fc*ɛ*RI in the plasma membrane of rat basophilic leukaemia (RBL)-2H3 cells. Their studies showed rapid decay in the mobility of Fc*ɛ*RI within a few seconds of stimulation with multivalent DNP-bovine serum albumin (DNP-BSA) antigens. By tracking individual receptor aggregates using super-resolution microscopy, Shelby *et al.* [16] showed about an order of magnitude decrease in mobility in 10–15 min (figure 4*a*). On the other hand, by tracking quantum dot (QD)-conjugated individual IgE-receptor complexes, Andrews *et al.* [16] reported about twofold decrease in the diffusion constant within a minute after stimulation (figure 4*b*). A direct and quantitative comparison or fitting between these data and our simulation results is not possible due to unknown parameters. Both studies used multivalent DNP-BSA ligands, whose exact structure and number of binding sites to interact with the receptors are unknown. On the other hand, we specifically modelled the structure of the trivalent ligand, DF3, reported in Mahajan *et al.* [8]. However, we were interested in investigating whether our model could predict similar rapid decay in post-stimulation diffusivity.

**Figure 5.**
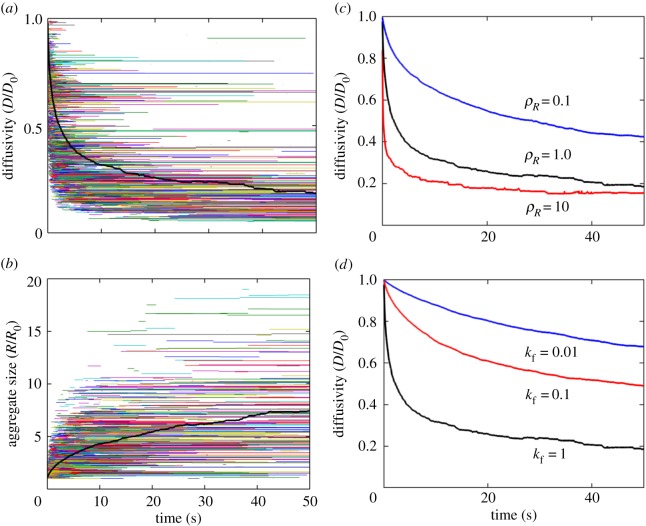
Temporal changes in species diffusion after stimulation. (*a*) Diffusion constants of individual receptor species (a single receptor molecule or a cluster). *D* and $D_{0}$ stand for the diffusion constants of an arbitrary receptor species and an isolated receptor molecule, respectively. Each horizontal line of a distinct colour represents a distinct receptor species of a finite lifetime. The thick dark line represents the mean diffusivity averaged over all the receptor species. (*b*) The sizes (effective radii) of individual receptor species are shown by the horizontal lines of distinct colours. *R* and $R_{0}$ stand for the effective radius of an arbitrary receptor species and a single isolated receptor molecule, respectively. The thick black line represents the mean size averaged over all receptor species. (*c*) Effect of receptor expression on the mean diffusivity. $\rho_{R}$ indicates receptor density normalized by the nominal density $n_{R}=80\;\mathrm{receptors}\;\mu m^{-2}$ (table 1). (*d*) Effect of the probability parameter *k*_f_ (table 1) on the mean diffusivity.

Figure 5*a* shows our model-predicted decay in the species diffusion after stimulation with the trivalent ligand. Figure 5*b* shows the corresponding time-evolution of the species sizes (effective radii). These results are obtained by using the default parameter values listed in table 1. In figure 5*a*, the diffusion constants of the time-evolving species are shown by the horizontal lines of distinct colours. The length of these lines indicate the lifetimes of the corresponding species. The start and end of each line indicate the time of appearance and disappearance of a species, respectively.

The mean diffusivity, which was obtained by averaging the diffusion constants of all species, indicates a rapid exponential decrease in mobility of the receptor aggregates immediately after stimulation. The result is consistent with the experimental observations in [14–16]. This rapid decrease is due to the fast growth of the initial species, which were relatively small and short-lived. The rate of decay eventually plateaued down as the larger aggregates populated at the expense of the more mobile smaller aggregates. The larger aggregates were relatively long-lived because they were less mobile and the system transitioned into a more dilute regime, as the interspecies distances became large.

We then investigated how receptor expression level may affect the dynamics (figure 5*c*). At low receptor densities, the rate of decay of diffusion became slower (figure 5*d*). This is expected because the larger interspecies distances in a dilute regime involved a less frequent encounter among the species. A reduced *k*_f_ also resulted in a slower decay in the diffusion (figure 5*d*). At a small *k*_f_, the system became more reaction-limited leading to many unproductive encounters among the species. It should be noted that, in our simulations, we considered monomeric Fc*ɛ*RI diffusion constant to be $10^{4}\;{\mathrm{nm}}^{2}\;s^{-1}$ [20]. However, Andrews *et al.* [14] reported Fc*ɛ*RI diffusion in unstimulated cells to be around $7\times 10^{4}\;{\mathrm{nm}}^{2}\;s^{-1}$. We used this value of diffusion constant to reproduce figure 5*d*. Corresponding figure is included in electronic supplementary material, figure S1). There was virtually no difference in the predictions with this new value of the diffusion constant (electronic supplementary material, figure S1).

### 3.3. Self-limiting receptor cross-linking and aggregation

The non-spatial models in the past indicated that Fc*ɛ*RI cross-linking by a trivalent ligand may lead to the superaggregate formation in the cell membrane [3,7]. We investigated to what extent the predictions of our spatial model might agree with the predictions of a non-spatial model. However, a direct comparison between the spatial and non-spatial model was not possible. In the spatial model, explicit diffusion and steric collisions determine receptor cross-linking. The non-spatial model lacks these features. In the non-spatial model, the association constant (*k*_on_) implicitly accounts for the diffusion and steric effects, as in any typical bimolecular reaction. This parameter is time-invariant and identical for all species regardless of their size or geometry.

**Figure 6.**
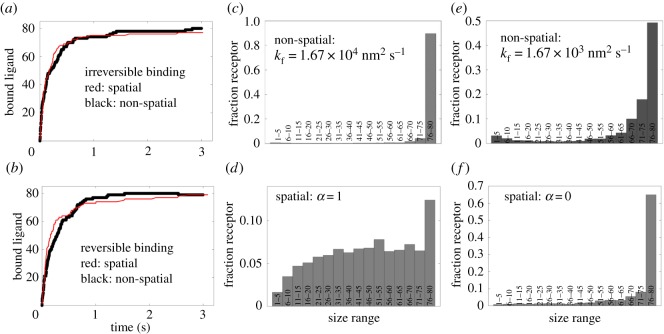
Comparison between the spatial and non-spatial model. (*a*) Amount of bound ligand (to receptors) versus time when ligand-receptor binding is irreversible in both models ($k_{r}=k_{\mathrm{off}}=0$). Red and black correspond to the non-spatial and spatial model, respectively. (*b*) The same as in (*a*) considering reversible binding ($k_{r}=k_{\mathrm{off}}=0.001\;s^{-1}$). (*c*) The non-spatial model-predicted receptor aggregate size distribution at *t* = 500 s. The distribution was created by grouping receptor aggregate sizes into different bins. Each bin indicates the range of aggregate sizes in terms of the number of receptor molecules. The *Y*-axis represents the normalized amount of receptors corresponding to each bin. The distribution represents samples from 1000 simulation runs. The simulations were performed using the default parameter values listed in table 2. (*d*) The spatial model-predicted receptor aggregate size distribution at *t* = 500 s. The distribution was created in the same way as in (*c*). The simulations were performed using the default parameter values listed in table 1. (*e*) The same as in (*c*) when *k*_f_ is reduced by 10-fold. (*f*) The same as in (*d*) when *α* = 0.

To make the spatial and non-spatial model more comparable, we first used the spatial model to predict the kinetics of ligand binding under two distinct conditions: irreversible binding ($k_{r}=0$) (figure 6*a*) and reversible binding ($k_{r}=0.001\;s^{-1}$) (figure 6*b*). We then fitted the non-spatial model to these predictions to evaluate *k*_on_ (figure 6*a*,*b*). We found the two models agreed well at $k_{\mathrm{on}}=1.67\times 10^{4}\;{\mathrm{nm}}^{2}\;s^{-1}$.

We then used both models to predict the distribution of receptor aggregates at *t* = 500 s. This simulation time was chosen to ensure that the mean distribution reached the steady-state condition. Similar to the earlier models [3,7], the non-spatial model predicted superaggregate formation where a single complex incorporated approximately all receptor molecules (figure 6*c*). By contrast, the spatial model predicted remarkably different distribution showing finite-sized receptor aggregation over a wide range of sizes (figure 6*d*). To check if the non-spatial model could also predict finite-sized aggregation at a lower *k*_on_, we reduced the parameter by 10-fold. However, the model still predicted superaggregate formation as shown in figure 6*e*. We found that the non-spatial model required approximately 100-fold reduction in *k*_on_ to predict finite-sized receptor aggregation (result not shown).

We then set *α* = 0 and allowed the spatial model to predict the distribution. At *α* = 0, the spatial model predicted superaggregate formation like the non-spatial model (figure 6*f*). In fact, at *α* = 0, the spatial model becomes more akin to the non-spatial model because the diffusivities of all species become time-invariant and identical. This is similar to the non-spatial model where the bimolecular association constant *k*_on_ is time-invariant and independent of the species.

**Figure 7.**
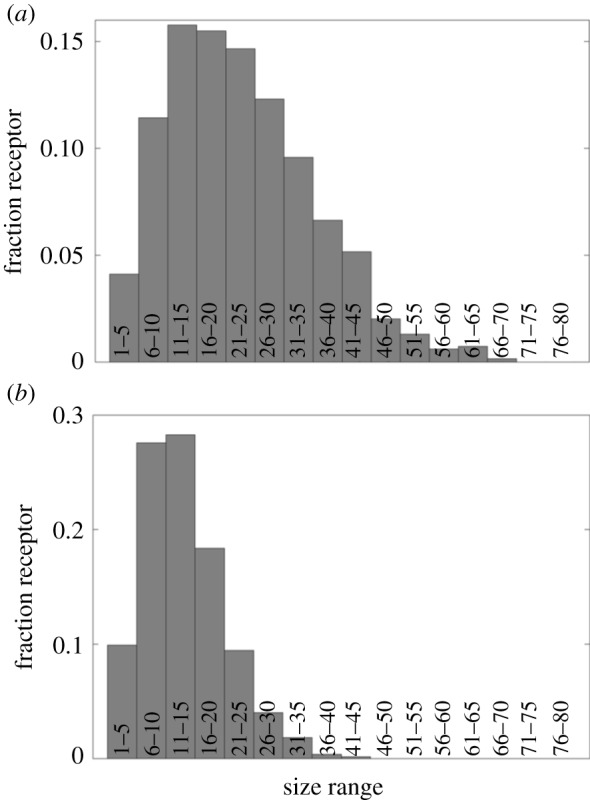
Self-limiting aggregation of receptors. The distributions correspond to simulation time *t* = s. (*a*) *α* = 2, (*b*) *α* = 3. Other parameter values used in the simulations are listed in table 1.

We next investigated the distribution of receptor aggregates at *α* > 1. An earlier experimental study by Menon *et al.* [25] demonstrated a dramatic loss of motion of Fc*ɛ*RI clusters containing more than two receptor proteins. Recently, Mahajan *et al.* [8] also reported a similar finding. These reports indicate that the diffusivity of the cross-linked receptors in the membrane may not exactly follow the natural size-dependence (*α* = 1). There might be anomalous effects giving rise to the sharp fall in the diffusivity of cross-linked receptors. In our model, we attempted to capture this effect by setting *α* > 1. Because $D\propto 1/R^{\alpha }$, *α* > 1 makes the diffusivity decay relatively faster. Figure 7*a*,*b* shows the distributions at *α* = 2 and *α* = 3, respectively. These values led to narrower distribution peaks and smaller receptor aggregate sizes when compared with the distribution in figure 6*d* (*α* = 1). In these analyses, the diffusion constant of monomeric Fc*ɛ*RI was set at the default value ($10^{4}\;{\mathrm{nm}}^{2}\;s^{-1}$). However, a significantly higher value reported in Andrews *et al.* [14] ($7\times 10^{4}\;{\mathrm{nm}}^{2}\;s^{-1}$) also did not change the result or conclusion (electronic supplementary material, figure S2).

It should be noted that, in our analysis, we treated the two ligand binding sites of the IgE-Fc*ɛ*RI as identical. However, the complex has an asymmetrical structure despite containing two identical chains [26]. It is unknown whether or to what extent such structural asymmetry makes a difference between the two sites in ligand binding. To our knowledge, all earlier models have treated the two sites as identical [3,7,8,27]. Our default condition was also set accordingly. However, to see if a mild difference between the two sites could change the predictions, we reduced the value of the intrinsic forward rate constant *k*_f_ by half for one of the two sites. This change virtually had no effect on the predictions. As shown in electronic supplementary material, figure S3, the model predicted finite-sized receptor aggregates identical to figure 6*d*. A significant reduction in *k*_f_ for one site would definitely make a difference because this would transform the receptor into a monomeric molecule, thus abolishing receptor cross-linking. However, since receptors do form aggregates via ligand-mediated cross-linking, such a scenario is unlikely. We should mention that a better strategy to consider the asymmetry would be to explicitly incorporate the structural topology of the IgE-Fc*ɛ*RI complex. However, this would require a three-dimensional extension of the spatial graphs, which is not within the scope of our current algorithm.

## 4. Discussion

Receptor aggregation and consequent reduction in their lateral diffusion in the cell membrane is a common phenomenon for many cell signalling receptor systems. Earlier, Schlessinger *et al.* [28,29] reported reduced diffusion of insulin growth factor receptor (IGFR) and epidermal growth factor receptor (EGFR) in response to stimulation by corresponding ligands, IGF and EGF, respectively [29]. Venkatakrishnan *et al.* [30] reported constitutive aggregation and reduced mobility of nerve growth factor receptors in the plasma membrane of medulloblastoma cells. T cell receptors (TCR) also form immobile microclusters during the formation of immunological synapse upon exposure to an antigen presenting cell (APC) [31,32]. The most extensively studied system in this regard is perhaps the Fc*ɛ*RI system, as evident from many experimental investigations spanning more than three decades [14–16,25,33–38]. Despite the numerous experimental investigations, however, very little theoretical or computational modelling works have been done to further gain insights into the consequences of such interrelationship between receptor aggregation and diffusion. To our knowledge, the first and perhaps the only modelling work in this context is the recent work of Mahajan *et al.* [8] who employed a non-spatial model to explain the phenomenon. The non-spatial model placed an implicit constraint that two receptor aggregates above a certain threshold size are unable to combine to form a larger aggregate. Because the model was non-spatial, this imposed constraint did not capture or explain the actual diffusion effect arising from the receptor aggregation in the membrane.

In this work, we used a spatio-temporal model to understand this relationship. Based on [8], we took Fc*ɛ*RI aggregation by a trivalent antigen as a model system for our analysis. However, the insights gained from the analysis may be generalized for other multivalent ligand-receptor systems as well. Multivalent ligand-mediated Fc*ɛ*RI aggregation is perhaps the most thoroughly studied problem in the context of the antigen–receptor interaction. Pioneering experimental works have been led by the Baird and Holowka laboratory and complementary modelling works have been done by scientists at the Los Alamos National Laboratory for decades [9,25,39,40]. Nonetheless, with the advent of new quantitative measurement tools and high-resolution microscopy techniques, many of these earlier works have been revisited [7,8,14–16].

Our study was primarily inspired by a caveat that most of the existing multivalent Fc*ɛ*RI aggregation models are non-spatial. This motivated us to develop the spatial model and investigate if incorporation of space could explain the experimentally observed phenomena in Fc*ɛ*RI aggregation. To the best of our knowledge, this work introduces the first spatio-temporal model of multivalent ligand-mediated Fc*ɛ*RI assembly in the cell membrane. The BD-based simulation approach used in the model is based on our recent work where we studied a generic multivalent ligand-receptor interaction [17]. The simulation approach explicitly accounts for the coarse-grained structure of the time-evolving species, their diffusion, and steric collisions.

An early model by Goldstein & Perelson [3] focused on equilibrium Fc*ɛ*RI aggregation in response to a trivalent ligand. This theoretical model was not capable of tracking the evolution of individual molecules or complexes. A later model by Monine *et al.* [7,41] allowed this capability by implementing a kinetic Monte Carlo (KMC) approach, called network-free simulation [22]. The network-free approach combined with the rule-based modelling (RBM) [23,42] provides a unique capability to model multivalent species interactions in signalling network system [41]. However, the models developed in this approach are non-spatial. Both the models in [2,21] indicated large Fc*ɛ*RI superaggregate formation in response to the trivalent antigen. The more recent model [8] was also developed using the non-spatial network-free KMC. However, the model prohibited superaggregate formation with the assumption that receptor clusters above a certain threshold size are immobile, whereas all clusters smaller than that threshold are equally mobile in the membrane.

While addressing the limitation of the non-spatial models above, our work provides important insights into the potential roles of membrane diffusion on immunoreceptor signalling. The key message from our analysis is that the membrane diffusion may limit receptor cross-linking and activation against stimulation by antigens. Earlier modelling studies postulated that Fc*ɛ*RI superaggregate formation may enable cells to become hyper-responsive against multivalent foreign antigens [3]. By contrast, our analysis in figures 6 and 7 indicates that the diffusion barriers limit receptor clustering, which may contain a cell from being hyper-responsive.

Similar to the earlier models, our model lacks receptor trafficking in the plasma membrane. We consider a fixed amount of Fc*ɛ*RI and we do not incorporate receptor synthesis, endocytosis, recycling or degradation. However, the potential effects of receptor trafficking in the model could be surmised. Receptor endocytosis might further put a restriction on receptor aggregation sizes. If receptors have a finite lifetime in the membrane, it would be less likely for the slowly diffusing receptor clusters to combine and form large aggregates. This may further diminish the possibility of a superaggregate formation. In our model, we analysed receptor size distribution at 500 s after stimulation. We did not see appreciable changes in the distributions at t>500 s even at *α* = 1. However, if infinite receptor lifetime is allowed in the absence of endocytosis and a sufficiently small rate for the bond dissociation is assumed, it is theoretically possible to form superaggregates in the spatial model.

We limited the scope of our model to the simple ligand-receptor system in two dimensions. The approach could be extended for spatio-temporal modelling of more complex signalling network systems. A three-dimensional extension of the approach might be required to more realistically model the extracellular and intracellular compartments of a cell. However, such an extension entails additional measures to address the computational challenges which were beyond the scope of this work. We believe that the predicted effects of diffusion on the receptor aggregation would remain similar if a three-dimensional membrane was considered in our model.

Currently, the RBM approach enables site-specific features and interactions of protein molecules. However, most of the RBM tools rely on non-spatial graphs to define these features. Recent efforts have been undertaken to enable spatio-temporal modelling in several rule-based tools, which include Kappa [43], Simmune [44] and BioNetGen [23]. Besides, there exist many stochastic modelling tools for spatio-temporal modelling with advanced capabilities [45]. Examples include MCell [46], Smoldyn [47], SPATKIN [48], ReaDDy [49] and SpringSaLaD [50]. However, to our knowledge, none of these tools have been used to create spatio-temporal models considering the site-specific coarse-grained structure of ligand and receptor molecules and their interactions.

Many of the above tools do not permit geometry or structure of molecules in a model. MCell [46] and Smoldyn [47] are two powerful tools that enable versatile capabilities to model reaction and diffusion in complex geometries (cellular or reaction compartments). However, both employ particle-based simulations where species are treated as points. Although Smoldyn can consider volume exclusion for isolated structures [51], to our knowledge, it does not permit time-evolving structures (geometries) arising from the site-specific binding of molecules or complexes, as implemented in our model.

The tools that might be closely related to our approach are SPATKIN [48], ReaDDy [49], and SpringSaLaD [50]. These software tools describe coarse-grained structures of species by spatial orientation of small beads, as in our model. However, SPATKIN allows lattice-based BD simulation. Because the lattice must be described *a priori*, unlike the time-adaptive feature implemented in our approach, such simulation could be expensive. The lattice must be very finely grained if the site-specific interaction of species is to be captured at a high resolution. Both ReaDDy and SpringSaLaD allow off-lattice BD. However, the simulation algorithm is distinct from the time-adaptive BD implemented in our approach. It is also unclear from the published literature if these tools permit time-evolving diffusion constant based on the evolving size and geometry of the species, an essential feature of our model. Nevertheless, the modelling and algorithmic features presented in our work may also be implemented in some of these tools without requiring any non-trivial modification in the software code.

## Supplementary Material

Supplementary Figures

## Supplementary Material

Simulation code: nonspatial model

## Supplementary Material

Simulation code: spatial model

## Data accessibility

The source codes for both spatial and non-spatial models are included in the electronic supplementary material (folders named ‘spatial_model.tar.gz’ and ‘nonspatial_model.tar.gz’, respectively). Detailed instructions for the installation and execution of simulations are provided in a file named ‘README.txt’ in each folder.
