# Supplementary material for: A spatio-temporal model reveals self-limiting FcɛRI cross-linking by multivalent antigens
Source: R Soc Open Sci. 2018 Sep 26;5(9):180190. doi: 10.1098/rsos.180190 (PMC6170560; doi:10.1098/rsos.180190)
Supplement: Supplementary Figures [file rsos180190supp1.pdf]

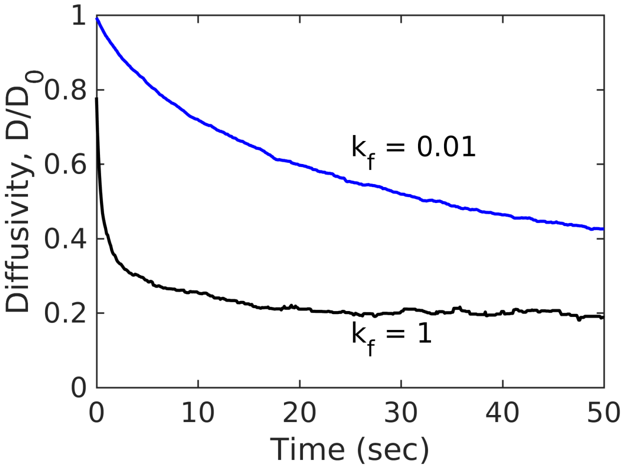

Figure S1. Time-dependent decay of mean species diffusivity. In the simulation, the monomeric receptor diffusivity is assumed to be 7×10^4 nm^2/s based on the reported value in Table 1 of Andrews et et al. (ref. 14 in main text). The plot shows how the mean diffusivity changes with time for two different values of the intrinsic forward rate constant k_f. The result is similar to Fig. 5D of the manuscript where the default diffusion constant value 10^4 nm^2/s was used for the monomeric receptors.

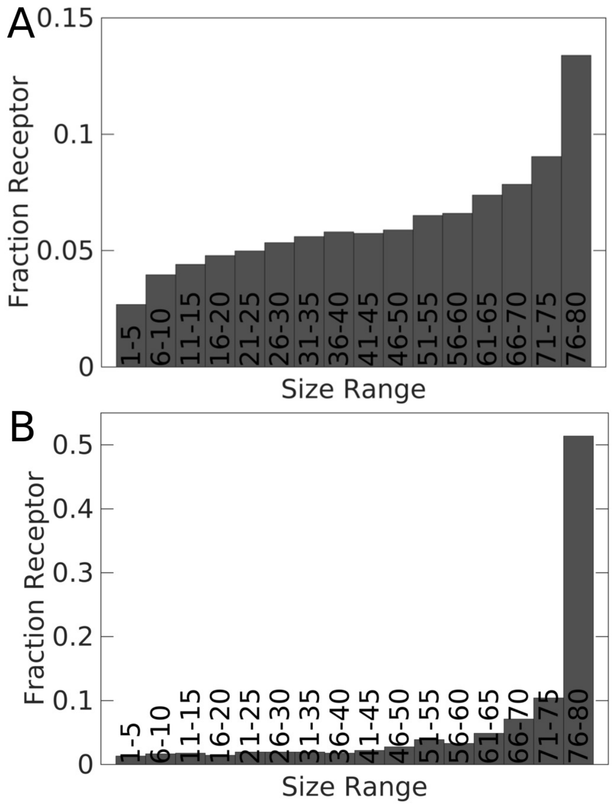

Figure S2. The spatial model predicted receptor aggregate size distribution for $\alpha = 1$ (Panel A) and $\alpha = 0$ (Panel B). Panel A represents the case where the diffusivity of ligand-receptor complexes naturally evolve based on the size of the complexes. Panel B represents the case where diffusivity is constant (equal to the monomeric receptor diffusivity) irrespective of the complex size. The panels correspond to Fig. 6D and Fig. 6F, respectively, where monomeric receptor diffusivity was 10^4 nm^2/s. Instead, these results are generated using monomeric receptor diffusivity 7×10^4 nm^2/s based on the reported value in Table 1 of Andrews et al. (ref. 14 in main text).

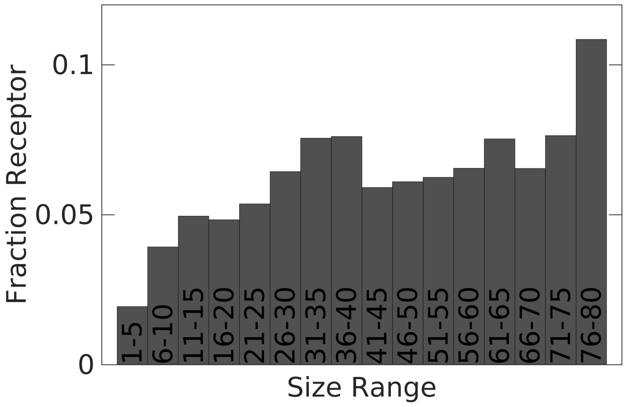

Figure S3. The spatial model predicted receptor aggregate size distribution when an asymmetry was incorporated in the two ligand binding sites of the receptor molecule. This asymmetry was incorporated considering distinct k_f values for the two binding sites (1 and 0.5 s^{-1}, respectively). Comparing this result with Fig. 6D, where both sites were assumed identical, this asymmetry did not lead to any noticeable difference.
